# Ca_V_ channels and cancer: canonical functions indicate benefits of repurposed drugs as cancer therapeutics

**DOI:** 10.1007/s00249-016-1144-z

**Published:** 2016-06-24

**Authors:** Paul J. Buchanan, Karen D. McCloskey

**Affiliations:** 1 Centre for Cancer Research and Cell Biology, School of Medicine, Dentistry and Biomedical Sciences, Queen’s University Belfast, 97 Lisburn Road, Belfast, Northern Ireland BT9 7AE UK; 2 National Institute of Cellular Biotechnology, School of Nursing and Human Science, Dublin City University, Glasnevin, Dublin 9, Ireland

**Keywords:** Cancer, Calcium channels, Repurposed drugs

## Abstract

The importance of ion channels in the hallmarks of many cancers is increasingly recognised. This article reviews current knowledge of the expression of members of the voltage-gated calcium channel family (Ca_V_) in cancer at the gene and protein level and discusses their potential functional roles. The ten members of the Ca_V_ channel family are classified according to expression of their pore-forming α-subunit; moreover, co-expression of accessory α2δ, β and γ confers a spectrum of biophysical characteristics including voltage dependence of activation and inactivation, current amplitude and activation/inactivation kinetics. Ca_V_ channels have traditionally been studied in excitable cells including neurones, smooth muscle, skeletal muscle and cardiac cells, and drugs targeting the channels are used in the treatment of hypertension and epilepsy. There is emerging evidence that several Ca_V_ channels are differentially expressed in cancer cells compared to their normal counterparts. Interestingly, a number of Ca_V_ channels also have non-canonical functions and are involved in transcriptional regulation of the expression of other proteins including potassium channels. Pharmacological studies show that Ca_V_ canonical function contributes to the fundamental biology of proliferation, cell-cycle progression and apoptosis. This raises the intriguing possibility that calcium channel blockers, approved for the treatment of other conditions, could be repurposed to treat particular cancers. Further research will reveal the full extent of both the canonical and non-canonical functions of Ca_V_ channels in cancer and whether calcium channel blockers are beneficial in cancer treatment.

## Introduction

Calcium signalling is an important physiological property of cells given the essential roles of calcium ions (Ca^2+^) in processes such as contraction, motility, apoptosis, transmitter release, exocytosis and endocytosis. Cells have many mechanisms for the precise regulation of intracellular Ca^2+^ concentration including ion channels [TRPs and voltage-gated calcium channels (VGCCs)], transporters and pumps on the plasma membrane and intracellular membranes, e.g., Na^+^/Ca^2+^ exchanger. VGCCs have been widely studied in the context of excitable cells in cardiovascular physiology, neuromuscular physiology and neuroscience, and their inhibition by several classes of calcium channel blockers (CCBs) is important in the treatment of hypertension and epilepsy.

Interestingly, VGCCs are functionally expressed in non-excitable cells including immune cells (Vig and Kinet 2009; Davenport et al. 2015) and a number of epithelial cancer cell types (Prevarskaya et al. 2010, 2014; Lastraioli et al. 2015; Wang et al. 2015). These cells also express TRP channels and it was considered that calcium signalling operated mainly via store-operated calcium channels, now defined molecularly as interactions between Orai channel proteins on the plasma membrane and STIM proteins on the endoplasmic reticulum (Soboloff et al. 2012; Hogan et al. 2010). It is now known that a diverse array of VGCCs is functionally active in non-excitable cells and contribute to Ca^2+^-dependent signalling processes. In cancer cells, VGCCs are involved in several of the cancer hallmarks, originally described by Hanahan and Weinberg (2000) as sustaining proliferative signalling, evading growth suppressors, resisting cell death, enabling replicative immortality, inducing angiogenesis, and activating invasion and metastasis and more recently updated (Hanahan and Weinberg 2011) to include the enabling hallmarks of reprogramming energy metabolism and evading immune destruction.

## Ca_V_ ion channel family

The VGCC family comprises ten members, based on expression of a specific pore-forming α_1_-subunit of 190–250 kDa containing the voltage sensor and binding sites for modulators and drugs and divided into three phylogenetic subfamilies: Ca_V_1, Ca_V_2 and Ca_V_3 (Ertel et al. 2000; Catterall 2011; Catterall et al. 2005; Alexander et al. 2015). In the approved nomenclature ‘Ca’ represents Ca^2+^ as the main permeating ion, ‘V’ indicating the physiological modulator, voltage and the number of the subfamily followed by its α1 subunit. The Ca_V_1 subfamily includes Ca_V_1.1 (α_1S_), Ca_V_1.2 (α_1C_), Ca_V_1.3 (α_1D_) and Ca_V_1.4 (α_1F_), known as L-type channels, describing ‘long-lasting currents’, which are typically high voltage-activated and dihydropyridine-sensitive. Ca_V_2.1, Ca_V_2.2 and Ca_V_2.3, are high voltage-activated and dihydropyridine-insensitive channels which contain α_1A_, α_1B_ and α_1E_ subunits, respectively, mediating P/Q-type, N-type and R-type Ca^2+^ currents. Ca_V_3 channels Ca_V_3.1 (α_1G_), Ca_V_3.2 (α_1H_) and Ca_V_3.3 (α_1I_) are low voltage-activated, dihydropyridine-sensitive, T-type or ‘transient currents’ indicating their kinetics of activation and inactivation (Catterall et al. 2005). L-type and T-type Ca_V_ families are expressed in many cell types while N, P/Q, and R-type channels are predominantly expressed in neurons. Alternative splicing of the pore-forming α subunits confers unique pharmacological and electrophysiological properties to VGCC representing remarkable plasticity and molecular diversity (Hofmann et al. 1994; Tan et al. 2011; Fan et al. 2005; Gray et al. 2007; Singh et al. 2008; Huang et al. 2013). It has been estimated that there are over 1000 theoretical splice isoforms from a single α1 subunit, based on known splice sites (Fox et al. 2008; Emerick et al. 2006; Gray et al. 2007).

### Accessory subunits

Activity of Ca_V_ channels is modulated by co-expression of a number of accessory subunits, α2δ, β and γ, which themselves have several members, α2δ1, α2δ2, α2δ3, α2δ4, β, β2, β3, β4 and γ. The emerging role of these subunits has recently been reviewed by Hofmann et al. (2015) and includes essential physiological processes of channel trafficking and stability in the plasma membrane in addition to regulation of channel activity.

Four mammalian genes have been identified for the α2δ1, α2δ2, α2δ3 and α2δ4 subunits, which are CACNA2D1, CACNA2D2, CACNA2D3 and CACNA2D4 respectively (Klugbauer et al. 2003; Davies et al. 2007). These subunits act to increase current amplitude through Ca_v_α_1_:Ca_v_β complexes, modify channel gating, induce a hyperpolarising shift in the voltage dependence of inactivation and modulate membrane stability in addition to being the binding sites for the anti-epileptic drugs gabapentin and pregabalin (Alexander et al. 2015).

Ca_V_β1-4 subunits are cytosolic proteins that act to regulate current density by controlling the amount of α_1_ subunit expressed at the cell membrane. In addition, β subunits regulate channel activation/inactivation kinetics and shift the voltage dependence of activation in the hyperpolarised direction. The Ca_V_γ subunits, of which eight have been identified, are structurally similar to the skeletal muscle Ca_V_γ1 subunit but γ2–8 are known to be transmembrane proteins involved in regulation of trafficking and gating of AMPA receptors and may not be linked with Ca_V_ channel activity (Hofmann et al. 2015).

### Electrophysiological properties

The Ca_V_ subfamilies have distinctive electrophysiological properties reflecting their molecular composition (see Catterall et al. 2005 for a comprehensive review). L-type Ca_V_1 currents typically activate positively to −40 mV, peak at 0 mV in physiological solutions and show voltage dependence of activation and inactivation. These long-lasting currents show slow calcium-dependent inactivation, which is absent when Ba^2+^ is the predominant charge carrier. Neuronal N-type Ca_V_2.2 channels have an intermediate voltage dependence and rate of inactivation that is faster than L-type and slower than T-type channels (Nowycky et al. 1985; Fox et al. 2008). T-type Ca_V_3 channels normally activate at more negative potentials than L-type, around −60 mV, peak at −20 mV and have faster kinetics of activation and inactivation.

### Pharmacology

Ca_V_1 and Ca_V_3 channels are sensitive to the dihydropyridine class of CCBs, which includes nifedipine, nimodipine, nisoldipine, felodipine and isradipine and can be activated by dihydropyridines such as Bay K8644. These drugs, rather than physically blocking the pore, act allosterically to shift the channel toward the open or closed state (Catterall et al. 2005). Within the Ca_V_1 and Ca_V_3 subfamilies, there is some differential sensitivity where Ca_V_1.2 is more sensitive to nifedipine than Ca_V_1.3 which is incompletely inhibited; moreover, Ca_V_3 channels are relatively less sensitive (Stengel et al. 1998). Phenylalkylamines, e.g., verapamil, are intracellular pore blockers, which are thought to enter the pore from the cytoplasmic side of the channel and cause occlusion (Catterall et al. 2005). Ca_V_1 channels are also sensitive to the CCB family of benzothiazepines such as diltiazem whereas Ca_V_2 and Ca_V_3 are not affected.

Ca_V_2.2 N-type currents are insensitive to dihydropyridines but can be blocked by the cone snail peptide ω-conotoxin GVIA and other related peptide toxins (Tsien et al. 1988; Olivera et al. 1994). Ca_V_2.1 P/Q currents have high sensitivity to the spider toxin ω-agatoxin IVA (Mintz et al. 1992) whereas Ca_V_ 2.3 R-type currents exhibit resistance to dihydropyridines but are sensitive to the tarantula-derived peptide SNX-482 (Newcomb et al. 1998).

Modulators of Ca_V_3 channels include Ni^2+^ ions and the small molecule inhibitor mibefradil, which is widely used (Martin et al. 2000; Lee et al. 1999). Ca_V_3.1 channels are blocked with kurtoxin, a peptide isolated from scorpion venom (Chuang et al.^1998^), although this is not selective as it also targets sodium channels. The diuretic amiloride blocks all Ca_V_3 families with varying affinities but has other targets including sodium channels (Tang et al. 1988; Lopez-Charcas et al. 2012; Zamponi et al. 2015).

## Expression of Ca_V_ in cancer

There is now compelling evidence that Ca_V_ channels are expressed in many cancers at the gene and protein level. Publically available data sets such as ONCOMINE are an excellent resource for investigation of ion channel expression from transcriptomic analyses across many studies. Wang et al. (2015) recently published a comprehensive meta-analysis of public microarray data sets reporting VGCC gene signatures from cancer patient samples demonstrating remarkable expression of Ca_V_ genes. A significant number of research groups have selected particular ion channels and characterised their functional expression with patch-clamp electrophysiology in primary cells derived from tumours or established cell lines.

### Ca_V_1.1

The Ca_V_1.1 gene (CACNA1S) is reportedly overexpressed in cancer compared with normal tissues in acute myeloid leukaemia, brain desmoplastic medulloblastoma and neuroectodermal tumours (Wang et al. 2015). Furthermore, this study revealed that CACNA1A was among the top 5 % upregulated genes in the leukaemia and brain cancer data sets. To date, the functional expression of Ca_V_1.1 in these cancers has not been investigated and this is potentially an exciting area of research.

### Ca_V_1.2

Overexpression of Ca_V_1.2 (gene CACNA1C) occurs in many cancers including colorectal, gastric, pancreatic, sarcoma, leukaemia, brain, breast, uterus, skin and prostate (Wang et al. 2015). This together with the finding that CACNA1C was in the top 10 % of upregulated genes may indicate involvement of Ca_V_1.2 in common molecular mechanisms of definitive carcinogenic events. In human breast cancer MCF7 cells, increased Ca_V_1.2 expression and reduced expression of the calcium binding protein, regucalcin, was induced by 5α-dihydrotestosterone (DHT), leading to reduced cell viability (Marques et al. 2015). Increased gene expression of CACNA1C has been reported for oesophageal squamous cell carcinoma, correlated with differentiation (Shi et al. 2015); however, there is no available information on protein expression or ion channel function. Similarly, a study of gene expression data from high-grade serous ovarian cancer patients showed 11 copy number amplified genes including CACNA1C (Davis et al. 2015). Wang et al. (2000) reported protein expression of CACNA1C, Ca_V_1.2α, in human colon cancer tissue samples and in Caco-2 and T84 cells. Interestingly, Western blotting and immunofluorescence revealed that Ca_V_1.2α was increased in non-confluent colonic cancer cells leading the authors to conclude that the functional expression of Ca_V_1.2α was linked with proliferation.

Calcium mobilisation via L-type calcium channels such as Ca_v_1.2 impacts other signalling mechanisms including activation of large-conductance, calcium-activated potassium channels, BK (Prakriya and Lingle 1999; Berkefeld et al. 2006). This is potentially important as BK channels are known to be involved in cancer biology (see the detailed review by Pardo and Stuhmer 2014) and Ca_V_1.2 therefore may indirectly drive cancer hallmarks by regulating BK channel activity.

### Ca_V_1.3

A study of the effects of nifedipine on endometrial carcinoma Hec-1A cells showed decreased proliferation and migration in addition to induction of autophagy via Beclin1 and mTOR pathways (Bao et al. 2012). The same group also reported expression of CACNA1D and CACNA1G in these cells and found that proliferation and migration were sensitive to mibefradil or nifedipine (Bao et al. 2012). Consistent with this, Ca_V_1.3 protein expression is reportedly higher in atypical hyperplasia and endometrial carcinoma tissues compared with benign endometrial tissue (Hao et al. 2015). Interestingly, Ca_V_1.3 expression was enhanced by 17β-estradiol and its knockdown reduced the 17β-estradiol-mediated stimulation of Ca^2+^ influx, proliferation and migration in endometrial cancer Ishikawa cells (Hao et al. 2015). Together, this work indicates the importance of Ca^2+^ influx via Ca_V_1.3 channels in endometrial carcinoma biology and further work should reveal the VGCC-related mechanisms underpinning this disease.

Electrophysiological patch-clamp experiments of MCF-7 breast cancer have demonstrated inward Ca^2+^ currents with the properties of both L and T-type Ca_V_ channels (Squecco et al. 2015). Pharmacological modulation with nifedipine or Ni^2+^ decreased cell proliferation confirming that Ca_V_ channels have physiological roles in breast cancer biology. Given that CACNA1C is known to be overexpressed in breast cancer tissue (Wang et al. 2015), it seems that Ca_V_1.2 and one or more of the Ca_V_3 subfamily are involved in the regulation of Ca^2+^ signalling in these cells.

Ca_V_1.3 is co-expressed with other Ca_V_ channels and their subunits in a number of cancers including neuronal neuroblastoma cells (Park et al. 2010; Chiou 2006; Grassi et al. 2004; Kito et al. 1999; Neelands et al. 2000). Sousa et al. (2013) detected transcripts for Ca_V_1.3, Ca_V_2.2, Ca_V_3.1 and a number of accessory subunits in SH-SY5Y human neuroblastoma cells. Depolarisation-induced Ca^2+^ events were nifedipine-sensitive and were also sensitive to the Ca_V_2.2 inhibitors ω-conotoxins CVID, GVIA and MVIIA demonstrating functional activity of the channels at the plasma membrane. Currents with the biophysical and pharmacological properties of L, N- and P/Q and R type Ca_V_ channels have been recorded in neuroblastoma cells (Neelands et al. 2000) showing that these cells functionally express a number of distinct channels that regulate Ca^2+^ signalling. The role of this panel of Ca_V_ channels in cancer hallmarks of neuroblastoma has not yet been determined and represents a promising area of research.

There is a compelling body of evidence that Ca_V_1.3 (CACNA1D) is overexpressed in prostate cancer at the gene and protein levels (Wang et al. 2015). Sun et al. (2006) reported that LNCaP prostate cancer cells displayed Ca^2+^ transients on stimulation with 5α-DHT and that these could be inhibited by the L-type channel inhibitors nifedipine, diltiazem or verapamil. A study of CACNA1D in prostate cancer in the ONCOMINE database (Chen et al. 2014a) revealed its significant overexpression in cancer tissues compared with normal prostate, consistent with findings of CACNAID mRNA and Ca_V_1.3 protein expression in prostate cancer cell lines. Interestingly the tumour microarray data showed that CACNA1D gene expression was higher in tumours with TMPRSS2-ERG fusion, which is in agreement with an epigenomic profiling study of prostate cancer tumours where CACNA1D was in the top-ranked differentially methylated genes in tissues with the TMPRSS2-ERG fusion (Geybels et al. 2015). Furthermore, other studies have reported CACNA1D to be in the top 10–20 genes most significantly correlated with ERG overexpression in patient tissues (Setlur et al. 2008; Jhavar et al. 2009; Boormans et al. 2013). In an evaluation of a panel of biomarkers to predict the aggressive prostate cancer phenotype, CACNA1D was correlated with Gleason score and biochemical recurrence (Zhu et al. 2015). Interestingly, high expression of CACNA1D was found to be an early event in active surveillance biopsies but in tumours with Gleason scores 4 + 3 or 8, CACNA1D was found to be lower. The significance of this finding needs to be investigated in other cohorts and experimental cell models.

Expression of Ca_V_1.3 in normal prostate cells is apparently very low, yet there is early evidence that these cells utilise Ca^2+^-signalling pathways, perhaps via other Ca_V_ channels. Connor et al. (1988) reported the dependence of hormonally induced prostate cell death on Ca^2+^-influx pathways; moreover, Martikainen and Isaacs (1990) found that Ca^2+^-dependent processes underpinned apoptosis induced by androgen removal.

It is interesting to note that the expression and function of Ca_V_1.2 and Ca_V_1.3 (discussed above) can be regulated by oestrogen and testosterone. This observation correlates with the high incidence of altered expression of these channels in the female and male reproductive systems (Table 1). Hormonal regulation of Ca_V_1 channels and the impact of this on their role in the development of particular cancers is a promising area for further investigation.

**Table 1:** Summary of tumour expression of voltage-gated calcium channel (Ca_V_) members and their associated functions to date

Ca_V_ member	Tumour expression	Function
Ca_V_1.1	Brain, leukaemia	Not defined
Ca_V_1.2	Brain, breast, colorectal, gastric, leukaemia, oesophageal, pancreatic, prostate, sarcoma, skin and uterine	Cell viability, proliferation, differentiation
Ca_V_1.3	Breast, neuroblastoma, prostate, uterine	Proliferation, migration
Ca_V_1.4	Testes	Not defined
Ca_V_2.1	Cervical, leukaemia, ovarian, brain, uterine, ovarian, lung	Growth progression
Ca_V_2.2	Breast, neuroblastoma, prostate	Not defined
Ca_V_2.3	Kidney, oesophageal, ovarian, pancreatic and uterine	Non-canonical progression, proliferation
Ca_V_3.1	Lung, pancreatic, neuroblastoma	Apoptotic resistance, autophagy, proliferation, cell cycle
Ca_V_3.2	Breast, leukaemia, glioblastoma, prostate	Apoptotic resistance, differentiation, proliferation, survival
Ca_V_3.3	Breast, colon, oesophageal, prostate, sarcoma	Proliferation

### Ca_V_1.4

There is limited information on the expression of Ca_V_1.4 (CACNA1F) in cancer. Mutations in CACNA1F cause the condition of incomplete congenital stationary night blindness (Striessnig et al. 2010). Wang et al. (2015) reported overexpression of CACAN1F in testicular teratoma in their meta-analysis of publically available TMA; functional expression of the Ca_V_1.4 protein in this or other tumours has not yet been reported.

### Ca_V_2.1

P/Q type channels are overexpressed in a number of cancer types including leukaemia, ovarian carcinoma, sarcoma, brain cancers, uterine corpus leiomyoma, ovarian cancer, lung cancers and cervical cancer (Wang et al. 2015). There is emerging evidence that these channels functionally contribute to cancer biology. Around 50–60 % of patients with Lambert-Eaton syndrome, an autoimmune disease characterised by production of autoimmune P/Q type antibodies (Titulaer et al. 2011a), go on to develop small-cell lung cancer. This neuroendocrine cancer is known to contain functional VGCCs (Titulaer et al. 2011b) and patients with small-cell lung cancer with low levels of P/Q antibodies had poor survival compared to those of Lambert-Eaton syndrome with high levels of the antibody, suggesting that increased function of Ca_V_2.1 P/Q channels may drive progression of the cancer (Roberts et al. 1985). This work also highlights the potential benefits of testing patients for the presence of these and other auto-immune antibodies in cancers that have known altered Ca_V_ expression or function.

Methylation of the CACNA1A gene is apparently associated with several cancers. In ovarian clear cell adenocarcinoma, increased methylation of CACNA1A was found to be linked with significantly reduced progression-free survival (Ho et al. 2012). Moreover, in lung cancer, CACNA1A has been identified as a novel tumour suppressor whose methylation was likely to result in adenocarcinoma (Castro et al. 2010). In contrast, non-methylation status of CACNA1A is reportedly associated with triple-negative breast cancer (Branham et al. 2012).

### Ca_V_2.2

CACNA1B is reported to be expressed in prostate and breast cancer (Wang et al. 2015). As discussed above, N-type Ca_V_2.2 channels are co-expressed with Ca_V_1 and Ca_V_3 channels in neuroblastoma cells (Kito et al. 1999; Sousa et al. 2013). A number of papers present pharmacological data supporting the functional expression of Ca_V_ 2.2 channels (Reeve et al. 1994; Reuveny and Narahashi 1993; Lambert et al. 1990; Andres et al. 2013; Morikawa et al. 1998; and Sher et al. 1996) although at the time of some of the earlier studies, it may not have been possible to determine the subtype expressed. Sousa et al. (2013) found three splice variants of Ca_V_2.2 in SH-SY5Y human neuroblastoma cells and in functional experiments observed effects from inhibition of Ca_V_2.2 channels.

### Ca_V_2.3

R-type channels (CACNA1E) are reported to be significantly overexpressed in oesophageal and uterine cancer (Wang et al. 2015) although data on functional expression are not yet available. CACNA1E is overexpressed in childhood kidney cancer Wilm’s tumours, predominantly in the nuclei, and is associated with increased risk of relapse (Natrajan et al. 2006). Overexpression of Ca_V_3.2 in HEK cells activated an MeK/ERK5/Nur77 pathway and may indicate a novel non-canonical role in cancer progression, which is a property of Ca_V_1.2 and Ca_V_1.3 channels (see below).

Ca_V_2.3 channels are also involved in FSH-stimulated ovarian cancer cell growth (Li et al. 2007), which occurs via a cAMP-independent activation of ERK and is sensitive to the Ca_V_2.3 channel inhibitor, SNX-482. Functional Ca_V_2.3 channels have been demonstrated in pancreatic cancer cells (Bon-1) where channel activity is coupled to IGF-1 signalling and secretion of chromogranin A (Mergler et al. 2003, 2005). A recent study of somatic mutations in patients with non-small-cell lung cancer exposed to severe air pollution compared with patients not exposed to pollution found that the most frequent mutations were related to calcium signalling, notably including CACNA1E (Yu et al. 2015). This finding shows that CACNA1E and potentially other Ca_V_ channel genes can acquire mutations and act as drivers for certain cancers in contrast to having altered expression as a consequence of mutations elsewhere.

### Ca_V_3.1

Choi et al. (2014) reported anti-proliferative and apoptotic activities of a T-type calcium channel antagonist, BK10040, in human lung adenocarcinoma (A549) and pancreatic cancer (MiaPaCa2) cells. Consistent with this, expression of CACNA1G in lung cancer has been reported by Wang et al. (2015). In a related pharmacological study, the putative T-channel blocker KYS05090 induced autophagy- and apoptosis-mediated cell death in human lung adenocarcinoma A549 cells (Rim et al. 2014) and while it decreased intracellular Ca^2+^ levels, it was not found to directly cause cell death. The authors reported generation of reactive oxygen species and reduced glucose uptake and while the drug may have potential in lung cancer treatment, it might be independent of Ca_V_3.1 activity. In a drug screening study on ovarian cancer cells, KYS05090 induced apoptosis, perhaps confirming the functional expression of T channels (Jang et al. 2013).

CACNA1G is highly expressed in human laryngeal squamous cell carcinoma tissues and experimental cell lines (Yu et al. 2014); moreover, siRNA techniques and the Ca_V_3 channel blocker mibefradil inhibited proliferation and arrested cell cycle progression. Lu et al. (2008) screened a panel of oesophageal cancer cell lines and found gene expression of Ca_V_3.1, Ca_V_3.2 and Ca_V_3.3; the latter also confirmed by Wang et al. (2015). Functional expression of T currents was confirmed by patch-clamp experiments and their role in cancer hallmarks was demonstrated by the reduction of proliferation by mibefradil or by siRNA. As discussed above, Ca_V_3.1 is co-expressed in SH-SY5Y human neuroblastoma cells along with other Ca_V_ channels and accessory subunits (Sousa et al. 2013).

### Ca_V_3.2

Neuroendocrine differentiation of prostate cancer cells is an important mechanism for the development of poor prognostic tumours and is known to involve increased expression of functional Ca_V_3.2 channels (Gackiere et al. 2008). In LNCaP cells, neuroendocrine differentiation evoked by androgen-reduced medium or cAMP increased the proportion of cells expressing Ca_V_3.2 channels (Weaver et al. 2015a; Mariot et al. 2002), which were characterised with patch clamp, pharmacological blockers and siRNA (Mariot et al. 2002). Ca_V_3.2 activity may act to stimulate secretion of mitogens and induce phenotypic change (Mariot et al. 2002; Fukami et al. 2015). It has also been shown that functional coupling between BK and Ca_V_3.2 channels may act to drive proliferation of prostate cancer cells (Gackiere et al. 2013). The involvement of the tumour microenvironment in the upregulation of Ca_V_3.2 in neuroendocrine differentiation is shown in recent work by Weaver et al. (2015b) where IL-6 significantly increased Ca_V_3.2 protein expression but did not affect mRNA expression, indicative of a post-transcriptional mechanism. Interestingly IL-6 alone did not increase the expression of functional channels in the membrane but co-stimulation by IL-6 and the cAMP agent (forskolin) did increase functional channel expression. The development of a neuroendocrine morphology was prevented by Ca_V_3.2 inhibition in IL-6-stimulated cells confirming the channels’ role in this phenotype.

Ca_V_ channels may also be functionally expressed in leukaemia and lymphoma cell lines. Mibefradil reduced cell growth via decreasing proliferation and promoting apoptosis linked with Ca^2+^ release from the endoplasmic reticulum (Huang et al. 2015) indicating that these channels also participate in haematological malignancies.

The breast cancer cell line MCF-7 expresses Ca_V_3.1 and Ca_V_3.2 (Taylor et al. 2008a; Ranzato et al. 2014; Squecco et al. 2015), which seem to be involved in proliferation. In live-cell Ca^2+^-imaging experiments, Ca_V_3 channel blockers inhibited Ca^2+^ transients confirming functional Ca^2+^ influx through these channels. Inhibition or knockdown of Ca_V_ channels inhibited MCF-7 proliferation but not that of non-cancer breast epithelial cells; moreover, gene expression of Ca_V_3.1 and Ca_V_3.2 was only found in rapidly growing non-confluent cells compared with confluent cells (Taylor et al. 2008b).

Ca_V_3.2 channel overexpression in glioblastoma multiform tumours is apparently associated with cell survival and resistance to therapy (Valerie et al. 2013). Inhibition or knockdown of Ca_V_3 channels was found to reduce cell viability and clonogenic survival and also induced apoptosis. Similar effects were not found with L-channel inhibition confirming that Ca_V_3.2 channels may represent novel targets for treatment of glioblastomas.

### Ca_V_3.3

There are few studies reporting CACNA1I in cancer; however, Wang et al. (2015) found overexpression of the gene in breast, sarcoma and oesophageal cancers. A study of colon, breast and prostate cancer cells subjected to increased extracellular pressure reported that T-type Ca_V_3.3 channels modulated pressure-stimulated proliferation in all of the cells studied (Basson et al. 2015).

## Non-canonical functions of Ca_V_1 channels

**Fig. 1:**
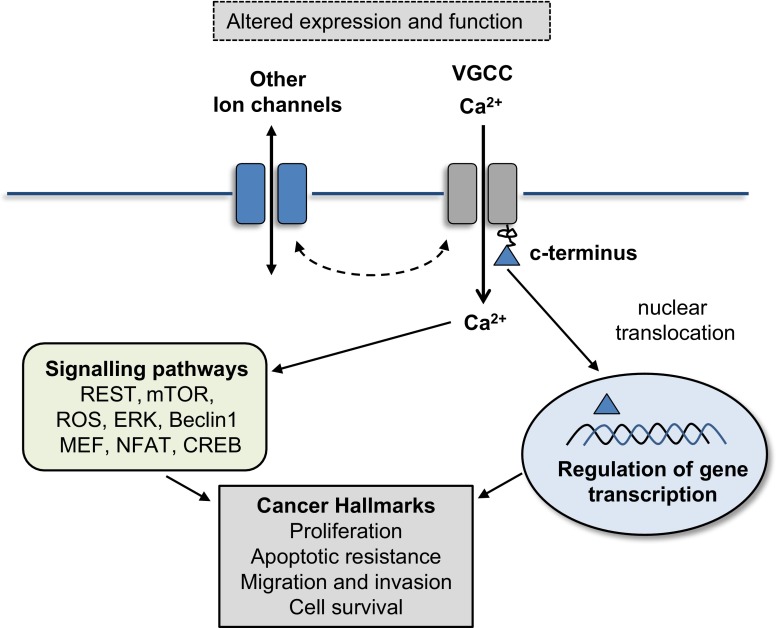
Schematic of potential mechanisms of Ca_V_ channels in cancer. In many cancers, expression and function of Ca_V_ channels is altered. There is compelling evidence that altered Ca_V_ expression and function contributes to several cancer hallmarks including proliferation, apoptotic resistance, migration, invasion and enhanced cell survival. These can arise through Ca^2+^-dependent signalling pathways via influx of Ca^2+^ through the Ca_V_ membrane channels. In addition, non-canonical signalling occurs, particularly in Ca_V_1.2 and Ca_V_1.3 where proteolytic cleavage of the c terminus produces a fragment that translocates to the nucleus and regulates the transcription of genes involved in processes of tumour development and progression

As highlighted above, in addition to transport of Ca^2+^, Ca_V_ channels also have non-canonical functions (Fig. 1). Ca_V_1.2 and Ca_V_1.3 α subunits have carboxyl terminus regions that can be cleaved not only modifying the remaining pore subunit (Gerhardstein et al. 2000; Gao et al. 2000, 2001) but also conferring functions in regulation of transcription when the c terminus translocates to the nucleus. Wei et al. (1994) found that removal of up to 70 % of the Ca_V_1.2 carboxyl terminus increased current density by facilitating coupling between voltage-dependent gating and channel opening, resulting in increased channel open probability. Cleavage of the c-terminus of Ca_V_1.2 has been shown to generate a transcription factor termed CCAT (Gomez-Ospina et al. 2006). Overexpression of the CCAT fragment resulted in altered expression of a number of proteins including the ion channels TRPV4 and KCNN3. Therefore, in addition to Ca^2+^ flux driving cancer-related signalling, the Ca_V_1.2 c-terminus can further contribute by modulating or recruiting the expression of other channels to drive a cancer phenotype. Cleavage of the Ca_V_1.3 c-terminus in atrial myocytes is reported to induce its translocation to the nucleus where it acts as a transcription factor, regulating the expression of SK2 channels (Lu et al. 2015). In addition, expression of a number of other proteins was altered including immunoglobulins, transcription factors and myosin light chain. Further work is required to define the genes regulated by actions of the c-terminus of VGCCs in different tissues and subsequent effects on cancer progression.

Interestingly, VGCCs are known to have a number of protein interaction sites along their amino acid sequence, which allows for interaction with proteins that influence gene transcription such as CREB, NFAT, calmodulin and MEK (see the review by Barbado et al. 2009), therefore enabling ion channels to indirectly control transcription of genes that are known to be involved in cancer development and progression (Xiao et al. 2010; Mancini and Toker 2009; Berchtold and Villalobo 2014).

## Potential of repurposing Ca_V_ drugs for cancer therapy

Given the functional expression of Ca_V_ channels in several cancers and their confirmed role in Ca^2+^ transport, the use of CCBs may be beneficial in treating the disease. Many of the CCBs used in experimental models are FDA approved for the treatment of hypertension, epilepsy, chronic pain, etc. Such drugs could potentially be repurposed to treat cancer; moreover, epidemiological evidence describes cancer risk in the context of CCB use for other conditions. Given the ability of CCBs to target multiple Ca_V_ channels, further pre-clinical research is required to determine whether an effect on in vivo tumours would occur.

A number of epidemiological studies have investigated whether CCBs confer benefits or disadvantages in cancer patient cohorts. Several investigations report that the use of CCBs for other conditions may be correlated with a reduced risk of prostate cancer (Debes et al. 2004; Fitzpatrick et al. 2001; Lever et al. 1998; Rodriguez et al. 2009). Furthermore, other investigations have shown that CCB use is associated with significantly reduced prostate tumour aggressiveness and development of advanced disease (Kemppainen et al. 2011; Poch et al. 2013). Similar studies in breast cancer (Saltzman et al. 2013) have yielded mixed reports of CCBs with one study showing a significant reduction in breast cancer risk (Fitzpatrick et al. 1997), another reporting a trend of risk reduction (Fryzek et al. 2006) and other studies reporting no association (Bergman et al. 2014; Li et al. 2003, 2013, 2014; Chen et al. 2014b, 2015; Devore et al. 2015). In colorectal, lung and colon cancer, a number of studies of CCBs have shown no beneficial correlation (Boudreau et al. 2008; Michels et al. 1998).

It is perhaps not surprising that the epidemiological data are not yet definitive as tissue cancers are a heterogeneous group of diseases with distinctive molecular subtypes. Stratification of patient data, e.g., in prostate cancer by the presence of the TMPRS2-ERG fusion status (Tomlins et al. 2008) where it is known that CACNA1D is highly overexpressed, may show benefits of CCB use prior to development of aggressive disease. If that were to be the case, the molecular pathological diagnosis of prostate tumours may be beneficial to stratify patients towards CCB treatment in addition to standard of care therapy.

The Ca_V_3 channel inhibitor, mibefradil, was previously FDA approved and used in clinical practice (Ertel and Clozel 1997) but was quickly withdrawn because of serious toxicity arising from effects on other transporters through adverse interactions with beta-blockers, digoxin, verapamil and diltiazem (Mullins et al. 1998). Other CCBs are well tolerated such as the diuretic amiloride, which also targets Ca_V_3 (Tang et al. 1988), and it may be beneficial in the treatment of cancers where Ca_V_3 is functionally overexpressed.

Drug repurposing could be enhanced by strategies that improve potency, selectivity and toxicity, for example the creation of a prodrug from linking the drug to a non-toxic promoiety (Karaman 2014). This strategy facilitates drug targeting to specific tissues with the benefits of reducing toxicity and improving selectivity by releasing the drug only at the target tissue site, e.g., tumour. A similar approach has been used with the sarcoplasmic/endoplasmic reticulum calcium adenosine triphosphatase (SERCA) pump inhibitor, thapsigargin, which has been linked to prostate-specific membrane antigen to direct it to prostate cancer cells (Denmeade et al. 2012). A similar approach could be used for drugs that target specific VGCCs that are expressed in tumour cells but that would have adverse effects in non-tumour cells. While this approach would take longer to reach clinical trial compared with simply repurposing existing drugs, advantages in reduced toxicity and better access to tumour cells would provide additional therapeutic benefits.

## Conclusions

The pre-clinical science and expression data reviewed here indicate that VGCCs are overexpressed in many cancers and that in the majority of cases these are functional channels, facilitating Ca^2+^ transport and homeostasis. The potential of CCBs in cancer treatment, in addition to chemotherapy, surgery and radiation therapy, has not yet been fully investigated through either prospective clinical trials or retrospective epidemiological cohort analysis. Repurposing of CCBs for the benefit of cancer patients therefore presents an attractive opportunity to improve human health.

## References

[CR1] Alexander SP, Catterall WA, Kelly E, Marrion N, Peters JA, Benson HE, Faccenda E, Pawson AJ, Sharman JL, Southan C, Davies JA, Collaborators CGTP (2015). The concise guide to PHARMACOLOGY 2015/16: voltage-gated ion channels. Br J Pharmacol.

[CR2] Andres D, Keyser BM, Petrali J, Benton B, Hubbard KS, McNutt PM, Ray R (2013). Morphological and functional differentiation in BE(2)-M17 human neuroblastoma cells by treatment with Trans-retinoic acid. BMC Neurosci.

[CR3] Bao XX, Xie BS, Li Q, Li XP, Wei LH, Wang JL (2012). Nifedipine induced autophagy through Beclin1 and mTOR pathway in endometrial carcinoma cells. Chin Med J (Engl).

[CR4] Barbado M, Fablet K, Ronjat M, De Waard M (2009). Gene regulation by voltage-dependent calcium channels. Biochim Biophys Acta.

[CR5] Basson MD, Zeng B, Downey C, Sirivelu MP, Tepe JJ (2015). Increased extracellular pressure stimulates tumor proliferation by a mechanosensitive calcium channel and PKC-beta. Mol Oncol.

[CR6] Berchtold MW, Villalobo A (2014). The many faces of calmodulin in cell proliferation, programmed cell death, autophagy, and cancer. Biochim Biophys Acta.

[CR7] Bergman GJ, Khan S, Danielsson B, Borg N (2014). Breast cancer risk and use of calcium channel blockers using Swedish population registries. JAMA Intern Med.

[CR8] Berkefeld H, Sailer CA, Bildl W, Rohde V, Thumfart JO, Eble S, Klugbauer N, Reisinger E, Bischofberger J, Oliver D, Knaus HG, Schulte U, Fakler B (2006). BKCa-Cav channel complexes mediate rapid and localized Ca2+-activated K+ signaling. Science.

[CR9] Boormans JL, Korsten H, Ziel-van der Made AJ, van Leenders GJ, de Vos CV, Jenster G, Trapman J (2013). Identification of TDRD1 as a direct target gene of ERG in primary prostate cancer. Int J Cancer.

[CR10] Boudreau DM, Koehler E, Rulyak SJ, Haneuse S, Harrison R, Mandelson MT (2008). Cardiovascular medication use and risk for colorectal cancer. Cancer Epidemiol Biomark Prev.

[CR11] Branham MT, Marzese DM, Laurito SR, Gago FE, Orozco JI, Tello OM, Vargas-Roig LM, Roque M (2012). Methylation profile of triple-negative breast carcinomas. Oncogenesis.

[CR12] Castro M, Grau L, Puerta P, Gimenez L, Venditti J, Quadrelli S, Sanchez-Carbayo M (2010). Multiplexed methylation profiles of tumor suppressor genes and clinical outcome in lung cancer. J Transl Med.

[CR13] Catterall WA (2011). Voltage-gated calcium channels. Cold Spring Harb Perspect Biol.

[CR14] Catterall WA, Perez-Reyes E, Snutch TP, Striessnig J (2005). International Union of Pharmacology. XLVIII. Nomenclature and structure–function relationships of voltage-gated calcium channels. Pharmacol Rev.

[CR15] Chen R, Zeng X, Zhang R, Huang J, Kuang X, Yang J, Liu J, Tawfik O, Thrasher JB, Li B (2014). Cav1.3 channel alpha1D protein is overexpressed and modulates androgen receptor transactivation in prostate cancers. Urol Oncol.

[CR16] Chen Q, Zhang Q, Zhong F, Guo S, Jin Z, Shi W, Chen C, He J (2014). Association between calcium channel blockers and breast cancer: a meta-analysis of observational studies. Pharmacoepidemiol Drug Saf.

[CR17] Chen L, Malone KE, Li CI (2015). Use of antihypertensive medications not associated with risk of contralateral breast cancer among women diagnosed with estrogen receptor-positive invasive breast cancer. Cancer Epidemiol Biomark Prev.

[CR18] Chiou WF (2006). Effect of Abeta exposure on the mRNA expression patterns of voltage-sensitive calcium channel alpha 1 subunits (alpha 1A-alpha 1D) in human SK-N-SH neuroblastoma. Neurochem Int.

[CR19] Choi DL, Jang SJ, Cho S, Choi HE, Rim HK, Lee KT, Lee JY (2014). Inhibition of cellular proliferation and induction of apoptosis in human lung adenocarcinoma A549 cells by T-type calcium channel antagonist. Bioorg Med Chem Lett.

[CR20] Chuang RS, Jaffe H, Cribbs L, Perez-Reyes E, Swartz KJ (1998). Inhibition of T-type voltage-gated calcium channels by a new scorpion toxin. Nat Neurosci.

[CR21] Connor J, Sawczuk IS, Benson MC, Tomashefsky P, O’Toole KM, Olsson CA, Buttyan R (1988). Calcium channel antagonists delay regression of androgen-dependent tissues and suppress gene activity associated with cell death. Prostate.

[CR22] Davenport B, Li Y, Heizer JW, Schmitz C, Perraud AL (2015). Signature channels of excitability no more: L-type channels in immune cells. Front Immunol.

[CR23] Davies A, Hendrich J, Van Minh AT, Wratten J, Douglas L, Dolphin AC (2007). Functional biology of the alpha(2)delta subunits of voltage-gated calcium channels. Trends Pharmacol Sci.

[CR24] Davis SJ, Sheppard KE, Anglesio MS, George J, Traficante N, Fereday S, Intermaggio MP, Menon U, Gentry-Maharaj A, Lubinski J, Gronwald J, Pearce CL, Pike MC, Wu A, Kommoss S, Pfisterer J, du Bois A, Hilpert F, Ramus SJ, Bowtell DD, Huntsman DG, Pearson RB, Simpson KJ, Campbell IG, Gorringe KL (2015). Enhanced GAB2 expression is associated with improved survival in high-grade serous ovarian cancer and sensitivity to PI3K inhibition. Mol Cancer Ther.

[CR25] Debes JD, Roberts RO, Jacobson DJ, Girman CJ, Lieber MM, Tindall DJ, Jacobsen SJ (2004). Inverse association between prostate cancer and the use of calcium channel blockers. Cancer Epidemiol Biomark Prev.

[CR26] Denmeade SR, Mhaka AM, Rosen DM, Brennen WN, Dalrymple S, Dach I, Olesen C, Gurel B, Demarzo AM, Wilding G, Carducci MA, Dionne CA, Moller JV, Nissen P, Christensen SB, Isaacs JT (2012). Engineering a prostate-specific membrane antigen-activated tumor endothelial cell prodrug for cancer therapy. Sci Transl Med.

[CR27] Devore EE, Kim S, Ramin CA, Wegrzyn LR, Massa J, Holmes MD, Michels KB, Tamimi RM, Forman JP, Schernhammer ES (2015). Antihypertensive medication use and incident breast cancer in women. Breast Cancer Res Treat.

[CR28] Emerick MC, Stein R, Kunze R, McNulty MM, Regan MR, Hanck DA, Agnew WS (2006). Profiling the array of Ca(v)3.1 variants from the human T-type calcium channel gene CACNA1G: alternative structures, developmental expression, and biophysical variations. Proteins.

[CR29] Ertel SI, Clozel JP (1997). Mibefradil (Ro 40-5967): the first selective T-type Ca2+ channel blocker. Expert Opin Investig Drugs.

[CR30] Ertel EA, Campbell KP, Harpold MM, Hofmann F, Mori Y, Perez-Reyes E, Schwartz A, Snutch TP, Tanabe T, Birnbaumer L, Tsien RW, Catterall WA (2000). Nomenclature of voltage-gated calcium channels. Neuron.

[CR31] Fan QI, Vanderpool KM, Chung HS, Marsh JD (2005). The L-type calcium channel alpha 1C subunit gene undergoes extensive, uncoordinated alternative splicing. Mol Cell Biochem.

[CR32] Fitzpatrick AL, Daling JR, Furberg CD, Kronmal RA, Weissfeld JL (1997). Use of calcium channel blockers and breast carcinoma risk in postmenopausal women. Cancer.

[CR33] Fitzpatrick AL, Daling JR, Furberg CD, Kronmal RA, Weissfeld JL (2001). Hypertension, heart rate, use of antihypertensives, and incident prostate cancer. Ann Epidemiol.

[CR34] Fox AP, Cahill AL, Currie KP, Grabner C, Harkins AB, Herring B, Hurley JH, Xie Z (2008). N- and P/Q-type Ca2+ channels in adrenal chromaffin cells. Acta Physiol (Oxf).

[CR35] Fryzek JP, Poulsen AH, Lipworth L, Pedersen L, Norgaard M, McLaughlin JK, Friis S (2006). A cohort study of antihypertensive medication use and breast cancer among Danish women. Breast Cancer Res Treat.

[CR36] Fukami K, Sekiguchi F, Yasukawa M, Asano E, Kasamatsu R, Ueda M, Yoshida S, Kawabata A (2015). Functional upregulation of the H2S/Cav3.2 channel pathway accelerates secretory function in neuroendocrine-differentiated human prostate cancer cells. Biochem Pharmacol.

[CR37] Gackiere F, Bidaux G, Delcourt P, Van Coppenolle F, Katsogiannou M, Dewailly E, Bavencoffe A, Van Chuoi-Mariot MT, Mauroy B, Prevarskaya N, Mariot P (2008). CaV3.2 T-type calcium channels are involved in calcium-dependent secretion of neuroendocrine prostate cancer cells. J Biol Chem.

[CR38] Gackiere F, Warnier M, Katsogiannou M, Derouiche S, Delcourt P, Dewailly E, Slomianny C, Humez S, Prevarskaya N, Roudbaraki M, Mariot P (2013). Functional coupling between large-conductance potassium channels and Cav3.2 voltage-dependent calcium channels participates in prostate cancer cell growth. Biol Open.

[CR39] Gao T, Bunemann M, Gerhardstein BL, Ma H, Hosey MM (2000). Role of the C terminus of the alpha 1C (CaV1.2) subunit in membrane targeting of cardiac L-type calcium channels. J Biol Chem.

[CR40] Gao T, Cuadra AE, Ma H, Bunemann M, Gerhardstein BL, Cheng T, Eick RT, Hosey MM (2001). C-terminal fragments of the alpha 1C (CaV1.2) subunit associate with and regulate L-type calcium channels containing C-terminal-truncated alpha 1C subunits. J Biol Chem.

[CR41] Gerhardstein BL, Gao T, Bunemann M, Puri TS, Adair A, Ma H, Hosey MM (2000). Proteolytic processing of the C terminus of the alpha(1C) subunit of L-type calcium channels and the role of a proline-rich domain in membrane tethering of proteolytic fragments. J Biol Chem.

[CR42] Geybels MS, Alumkal JJ, Luedeke M, Rinckleb A, Zhao S, Shui IM, Bibikova M, Klotzle B, van den Brandt PA, Ostrander EA, Fan JB, Feng Z, Maier C, Stanford JL (2015). Epigenomic profiling of prostate cancer identifies differentially methylated genes in TMPRSS2:ERG fusion-positive versus fusion-negative tumors. Clin Epigenet.

[CR43] Gomez-Ospina N, Tsuruta F, Barreto-Chang O, Hu L, Dolmetsch R (2006). The C terminus of the L-type voltage-gated calcium channel Ca(V)1.2 encodes a transcription factor. Cell.

[CR44] Grassi C, D’Ascenzo M, Torsello A, Martinotti G, Wolf F, Cittadini A, Azzena GB (2004). Effects of 50 Hz electromagnetic fields on voltage-gated Ca2+ channels and their role in modulation of neuroendocrine cell proliferation and death. Cell Calcium.

[CR45] Gray AC, Raingo J, Lipscombe D (2007). Neuronal calcium channels: splicing for optimal performance. Cell Calcium.

[CR46] Hanahan D, Weinberg RA (2000). The hallmarks of cancer. Cell.

[CR47] Hanahan D, Weinberg RA (2011). Hallmarks of cancer: the next generation. Cell.

[CR48] Hao J, Bao X, Jin B, Wang X, Mao Z, Li X, Wei L, Shen D, Wang JL (2015). Ca2+ channel subunit alpha 1D promotes proliferation and migration of endometrial cancer cells mediated by 17beta-estradiol via the G protein-coupled estrogen receptor. Faseb j.

[CR49] Ho CM, Huang CJ, Huang CY, Wu YY, Chang SF, Cheng WF (2012). Promoter methylation status of HIN-1 associated with outcomes of ovarian clear cell adenocarcinoma. Mol Cancer.

[CR50] Hofmann F, Biel M, Flockerzi V (1994). Molecular basis for Ca2+ channel diversity. Annu Rev Neurosci.

[CR51] Hofmann F, Belkacemi A, Flockerzi V (2015). Emerging alternative functions for the auxiliary subunits of the voltage-gated calcium channels. Curr Mol Pharmacol.

[CR52] Hogan PG, Lewis RS, Rao A (2010). Molecular basis of calcium signaling in lymphocytes: STIM and ORAI. Annu Rev Immunol.

[CR53] Huang H, Yu D, Soong TW (2013). C-terminal alternative splicing of CaV1.3 channels distinctively modulates their dihydropyridine sensitivity. Mol Pharmacol.

[CR54] Huang W, Lu C, Wu Y, Ouyang S, Chen Y (2015). T-type calcium channel antagonists, mibefradil and NNC-55-0396 inhibit cell proliferation and induce cell apoptosis in leukemia cell lines. J Exp Clin Cancer Res.

[CR55] Jang SJ, Choi HW, Choi DL, Cho S, Rim HK, Choi HE, Kim KS, Huang M, Rhim H, Lee KT, Lee JY (2013). In vitro cytotoxicity on human ovarian cancer cells by T-type calcium channel blockers. Bioorg Med Chem Lett.

[CR56] Jhavar S, Brewer D, Edwards S, Kote-Jarai Z, Attard G, Clark J, Flohr P, Christmas T, Thompson A, Parker M, Shepherd C, Stenman UH, Marchbank T, Playford RJ, Woodhouse C, Ogden C, Fisher C, Kovacs G, Corbishley C, Jameson C, Norman A, De-Bono J, Bjartell A, Eeles R, Cooper CS (2009). Integration of ERG gene mapping and gene-expression profiling identifies distinct categories of human prostate cancer. BJU Int.

[CR57] Karaman R (2014). Using predrugs to optimize drug candidates. Expert Opin Drug Discov.

[CR58] Kemppainen KJ, Tammela TL, Auvinen A, Murtola TJ (2011). The association between antihypertensive drug use and incidence of prostate cancer in Finland: a population-based case-control study. Cancer Causes Control.

[CR59] Kito M, Maehara M, Watanabe K (1999). Three types of voltage-dependent calcium currents developing in cultured human neuroblastoma cells. Nagoya J Med Sci.

[CR60] Klugbauer N, Marais E, Hofmann F (2003). Calcium channel alpha2delta subunits: differential expression, function, and drug binding. J Bioenerg Biomembr.

[CR61] Lambert DG, Whitham EM, Baird JG, Nahorski SR (1990). Different mechanisms of Ca2+ entry induced by depolarization and muscarinic receptor stimulation in SH-SY5Y human neuroblastoma cells. Brain Res Mol Brain Res.

[CR62] Lastraioli E, Iorio J, Arcangeli A (2015). Ion channel expression as promising cancer biomarker. Biochim Biophys Acta.

[CR63] Lee JH, Gomora JC, Cribbs LL, Perez-Reyes E (1999). Nickel block of three cloned T-type calcium channels: low concentrations selectively block alpha1H. Biophys J.

[CR64] Lever AF, Hole DJ, Gillis CR, McCallum IR, McInnes GT, MacKinnon PL, Meredith PA, Murray LS, Reid JL, Robertson JW (1998). Do inhibitors of angiotensin-I-converting enzyme protect against risk of cancer?. Lancet.

[CR65] Li CI, Malone KE, Weiss NS, Boudreau DM, Cushing-Haugen KL, Daling JR (2003). Relation between use of antihypertensive medications and risk of breast carcinoma among women ages 65–79 years. Cancer.

[CR66] Li Y, Ganta S, Cheng C, Craig R, Ganta RR, Freeman LC (2007). FSH stimulates ovarian cancer cell growth by action on growth factor variant receptor. Mol Cell Endocrinol.

[CR67] Li CI, Daling JR, Tang MT, Haugen KL, Porter PL, Malone KE (2013). Use of antihypertensive medications and breast cancer risk among women aged 55 to 74 years. JAMA Intern Med.

[CR68] Li W, Shi Q, Wang W, Liu J, Li Q, Hou F (2014). Calcium channel blockers and risk of breast cancer: a meta-analysis of 17 observational studies. PLoS One.

[CR69] Lopez-Charcas O, Rivera M, Gomora JC (2012). Block of human CaV3 channels by the diuretic amiloride. Mol Pharmacol.

[CR70] Lu F, Chen H, Zhou C, Liu S, Guo M, Chen P, Zhuang H, Xie D, Wu S (2008). T-type Ca2+ channel expression in human esophageal carcinomas: a functional role in proliferation. Cell Calcium.

[CR71] Lu L, Sirish P, Zhang Z, Woltz RL, Li N, Timofeyev V, Knowlton AA, Zhang XD, Yamoah EN, Chiamvimonvat N (2015). Regulation of gene transcription by voltage-gated L-type calcium channel, Cav1.3. J Biol Chem.

[CR72] Mancini M, Toker A (2009). NFAT proteins: emerging roles in cancer progression. Nat Rev Cancer.

[CR73] Mariot P, Vanoverberghe K, Lalevee N, Rossier MF, Prevarskaya N (2002). Overexpression of an alpha 1H (Cav3.2) T-type calcium channel during neuroendocrine differentiation of human prostate cancer cells. J Biol Chem.

[CR74] Marques R, Peres CG, Vaz CV, Gomes IM, Figueira MI, Cairrao E, Verde I, Maia CJ, Socorro S (2015). 5alpha-Dihydrotestosterone regulates the expression of L-type calcium channels and calcium-binding protein regucalcin in human breast cancer cells with suppression of cell growth. Med Oncol.

[CR75] Martikainen P, Isaacs J (1990). Role of calcium in the programmed death of rat prostatic glandular cells. Prostate.

[CR76] Martin RL, Lee JH, Cribbs LL, Perez-Reyes E, Hanck DA (2000). Mibefradil block of cloned T-type calcium channels. J Pharmacol Exp Ther.

[CR77] Mergler S, Wiedenmann B, Prada J (2003). R-type Ca(2+)-channel activity is associated with chromogranin A secretion in human neuroendocrine tumor BON cells. J Membr Biol.

[CR78] Mergler S, Strauss O, Strowski M, Prada J, Drost A, Langrehr J, Neuhaus P, Wiedenmann B, Ploeckinger U (2005). Insulin-like growth factor-1 increases intracellular calcium concentration in human primary neuroendocrine pancreatic tumor cells and a pancreatic neuroendocrine tumor cell line (BON-1) via R-type Ca2+ channels and regulates chromogranin a secretion in BON-1 cells. Neuroendocrinology.

[CR79] Michels KB, Rosner BA, Walker AM, Stampfer MJ, Manson JE, Colditz GA, Hennekens CH, Willett WC (1998). Calcium channel blockers, cancer incidence, and cancer mortality in a cohort of U.S. women: the nurses’ health study. Cancer.

[CR80] Mintz IM, Venema VJ, Swiderek KM, Lee TD, Bean BP, Adams ME (1992). P-type calcium channels blocked by the spider toxin omega-Aga-IVA. Nature.

[CR81] Morikawa H, Fukuda K, Mima H, Shoda T, Kato S, Mori K (1998). Nociceptin receptor-mediated Ca2+ channel inhibition and its desensitization in NG108-15 cells. Eur J Pharmacol.

[CR82] Mullins ME, Horowitz BZ, Linden DH, Smith GW, Norton RL, Stump J (1998). Life-threatening interaction of mibefradil and beta-blockers with dihydropyridine calcium channel blockers. JAMA.

[CR83] Natrajan R, Little SE, Reis-Filho JS, Hing L, Messahel B, Grundy PE, Dome JS, Schneider T, Vujanic GM, Pritchard-Jones K, Jones C (2006). Amplification and overexpression of CACNA1E correlates with relapse in favorable histology Wilms' tumors. Clin Cancer Res.

[CR84] Neelands TR, King AP, Macdonald RL (2000). Functional expression of L-, N-, P/Q-, and R-type calcium channels in the human NT2-N cell line. J Neurophysiol.

[CR85] Newcomb R, Szoke B, Palma A, Wang G, Chen X, Hopkins W, Cong R, Miller J, Urge L, Tarczy-Hornoch K, Loo JA, Dooley DJ, Nadasdi L, Tsien RW, Lemos J, Miljanich G (1998). Selective peptide antagonist of the class E calcium channel from the venom of the tarantula *Hysterocrates gigas*. Biochemistry.

[CR86] Nowycky MC, Fox AP, Tsien RW (1985). Three types of neuronal calcium channel with different calcium agonist sensitivity. Nature.

[CR87] Olivera BM, Miljanich GP, Ramachandran J, Adams ME (1994). Calcium channel diversity and neurotransmitter release: the omega-conotoxins and omega-agatoxins. Annu Rev Biochem.

[CR88] Pardo LA, Stuhmer W (2014). The roles of K(+) channels in cancer. Nat Rev Cancer.

[CR89] Park JH, Park SJ, Chung MK, Jung KH, Choi MR, Kim Y, Chai YG, Kim SJ, Park KS (2010). High expression of large-conductance Ca2+-activated K+ channel in the CD133+ subpopulation of SH-SY5Y neuroblastoma cells. Biochem Biophys Res Commun.

[CR90] Poch MA, Mehedint D, Green DJ, Payne-Ondracek R, Fontham ET, Bensen JT, Attwood K, Wilding GE, Guru KA, Underwood W, Mohler JL, Heemers HV (2013). The association between calcium channel blocker use and prostate cancer outcome. Prostate.

[CR91] Prakriya M, Lingle CJ (1999). BK channel activation by brief depolarizations requires Ca2+ influx through L- and Q-type Ca2+ channels in rat chromaffin cells. J Neurophysiol.

[CR92] Prevarskaya N, Skryma R, Shuba Y (2010). Ion channels and the hallmarks of cancer. Trends Mol Med.

[CR93] Prevarskaya N, Ouadid-Ahidouch H, Skryma R, Shuba Y (2014). Remodelling of Ca2+ transport in cancer: how it contributes to cancer hallmarks?. Philos Trans R Soc Lond B Biol Sci.

[CR94] Ranzato E, Magnelli V, Martinotti S, Waheed Z, Cain SM, Snutch TP, Marchetti C, Burlando B (2014). Epigallocatechin-3-gallate elicits Ca2+ spike in MCF-7 breast cancer cells: essential role of Cav3.2 channels. Cell Calcium.

[CR95] Reeve HL, Vaughan PF, Peers C (1994). Calcium channel currents in undifferentiated human neuroblastoma (SH-SY5Y) cells: actions and possible interactions of dihydropyridines and omega-conotoxin. Eur J Neurosci.

[CR96] Reuveny E, Narahashi T (1993). Two types of high voltage-activated calcium channels in SH-SY5Y human neuroblastoma cells. Brain Res.

[CR97] Rim HK, Cho S, Shin DH, Chung KS, Cho YW, Choi JH, Lee JY, Lee KT (2014). T-type Ca2+ channel blocker, KYS05090 induces autophagy and apoptosis in A549 cells through inhibiting glucose uptake. Molecules.

[CR98] Roberts A, Perera S, Lang B, Vincent A, Newsom-Davis J (1985). Paraneoplastic myasthenic syndrome IgG inhibits 45Ca2+ flux in a human small cell carcinoma line. Nature.

[CR99] Rodriguez C, Jacobs EJ, Deka A, Patel AV, Bain EB, Thun MJ, Calle EE (2009). Use of blood-pressure-lowering medication and risk of prostate cancer in the Cancer Prevention Study II Nutrition Cohort. Cancer Causes Control.

[CR100] Saltzman BS, Weiss NS, Sieh W, Fitzpatrick AL, McTiernan A, Daling JR, Li CI (2013). Use of antihypertensive medications and breast cancer risk. Cancer Causes Control.

[CR101] Setlur SR, Mertz KD, Hoshida Y, Demichelis F, Lupien M, Perner S, Sboner A, Pawitan Y, Andren O, Johnson LA, Tang J, Adami HO, Calza S, Chinnaiyan AM, Rhodes D, Tomlins S, Fall K, Mucci LA, Kantoff PW, Stampfer MJ, Andersson SO, Varenhorst E, Johansson JE, Brown M, Golub TR, Rubin MA (2008). Estrogen-dependent signaling in a molecularly distinct subclass of aggressive prostate cancer. J Natl Cancer Inst.

[CR102] Sher E, Cesare P, Codignola A, Clementi F, Tarroni P, Pollo A, Magnelli V, Carbone E (1996). Activation of delta-opioid receptors inhibits neuronal-like calcium channels and distal steps of Ca(2+)-dependent secretion in human small-cell lung carcinoma cells. J Neurosci.

[CR103] Shi ZZ, Shang L, Jiang YY, Shi F, Xu X, Wang MR, Hao JJ (2015). Identification of genomic biomarkers associated with the clinicopathological parameters and prognosis of esophageal squamous cell carcinoma. Cancer Biomark.

[CR104] Singh A, Gebhart M, Fritsch R, Sinnegger-Brauns MJ, Poggiani C, Hoda JC, Engel J, Romanin C, Striessnig J, Koschak A (2008). Modulation of voltage- and Ca2+-dependent gating of CaV1.3 L-type calcium channels by alternative splicing of a C-terminal regulatory domain. J Biol Chem.

[CR105] Soboloff J, Rothberg BS, Madesh M, Gill DL (2012). STIM proteins: dynamic calcium signal transducers. Nat Rev Mol Cell Biol.

[CR106] Sousa SR, Vetter I, Ragnarsson L, Lewis RJ (2013). Expression and pharmacology of endogenous Cav channels in SH-SY5Y human neuroblastoma cells. PLoS One.

[CR107] Squecco R, Tani A, Zecchi-Orlandini S, Formigli L, Francini F (2015). Melatonin affects voltage-dependent calcium and potassium currents in MCF-7 cell line cultured either in growth or differentiation medium. Eur J Pharmacol.

[CR108] Stengel W, Jainz M, Andreas K (1998). Different potencies of dihydropyridine derivatives in blocking T-type but not L-type Ca2+ channels in neuroblastoma-glioma hybrid cells. Eur J Pharmacol.

[CR109] Striessnig J, Bolz HJ, Koschak A (2010). Channelopathies in Cav1.1, Cav1.3, and Cav1.4 voltage-gated L-type Ca2+ channels. Pflugers Arch.

[CR110] Sun YH, Gao X, Tang YJ, Xu CL, Wang LH (2006). Androgens induce increases in intracellular calcium via a G protein-coupled receptor in LNCaP prostate cancer cells. J Androl.

[CR111] Tan BZ, Jiang F, Tan MY, Yu D, Huang H, Shen Y, Soong TW (2011). Functional characterization of alternative splicing in the C terminus of L-type CaV1.3 channels. J Biol Chem.

[CR112] Tang CM, Presser F, Morad M (1988). Amiloride selectively blocks the low threshold (T) calcium channel. Science.

[CR113] Taylor JT, Huang L, Pottle JE, Liu K, Yang Y, Zeng X, Keyser BM, Agrawal KC, Hansen JB, Li M (2008). Selective blockade of T-type Ca2+ channels suppresses human breast cancer cell proliferation. Cancer Lett.

[CR114] Taylor JT, Zeng XB, Pottle JE, Lee K, Wang AR, Yi SG, Scruggs JA, Sikka SS, Li M (2008). Calcium signaling and T-type calcium channels in cancer cell cycling. World J Gastroenterol.

[CR115] Titulaer MJ, Lang B, Verschuuren JJ (2011). Lambert–Eaton myasthenic syndrome: from clinical characteristics to therapeutic strategies. Lancet Neurol.

[CR116] Titulaer MJ, Maddison P, Sont JK, Wirtz PW, Hilton-Jones D, Klooster R, Willcox N, Potman M, Sillevis Smitt PA, Kuks JB, Roep BO, Vincent A, van der Maarel SM, van Dijk JG, Lang B, Verschuuren JJ (2011). Clinical Dutch–English Lambert–Eaton myasthenic syndrome (LEMS) tumor association prediction score accurately predicts small-cell lung cancer in the LEMS. J Clin Oncol.

[CR117] Tomlins SA, Laxman B, Varambally S, Cao X, Yu J, Helgeson BE, Cao Q, Prensner JR, Rubin MA, Shah RB, Mehra R, Chinnaiyan AM (2008). Role of the TMPRSS2-ERG gene fusion in prostate cancer. Neoplasia.

[CR118] Tsien RW, Lipscombe D, Madison DV, Bley KR, Fox AP (1988). Multiple types of neuronal calcium channels and their selective modulation. Trends Neurosci.

[CR119] Valerie NC, Dziegielewska B, Hosing AS, Augustin E, Gray LS, Brautigan DL, Larner JM, Dziegielewski J (2013). Inhibition of T-type calcium channels disrupts Akt signaling and promotes apoptosis in glioblastoma cells. Biochem Pharmacol.

[CR120] Vig M, Kinet JP (2009). Calcium signaling in immune cells. Nat Immunol.

[CR121] Wang XT, Nagaba Y, Cross HS, Wrba F, Zhang L, Guggino SE (2000). The mRNA of L-type calcium channel elevated in colon cancer: protein distribution in normal and cancerous colon. Am J Pathol.

[CR122] Wang CY, Lai MD, Phan NN, Sun Z, Lin YC (2015). Meta-analysis of public microarray datasets reveals voltage-gated calcium gene signatures in clinical cancer patients. PLoS One.

[CR123] Weaver EM, Zamora FJ, Hearne JL, Martin-Caraballo M (2015). Posttranscriptional regulation of T-type Ca(2+) channel expression by interleukin-6 in prostate cancer cells. Cytokine.

[CR124] Weaver EM, Zamora FJ, Puplampu-Dove YA, Kiessu E, Hearne JL, Martin-Caraballo M (2015). Regulation of T-type calcium channel expression by sodium butyrate in prostate cancer cells. Eur J Pharmacol.

[CR125] Wei X, Neely A, Lacerda AE, Olcese R, Stefani E, Perez-Reyes E, Birnbaumer L (1994). Modification of Ca2+ channel activity by deletions at the carboxyl terminus of the cardiac alpha 1 subunit. J Biol Chem.

[CR126] Xiao X, Li BX, Mitton B, Ikeda A, Sakamoto KM (2010). Targeting CREB for cancer therapy: friend or foe. Curr Cancer Drug Targets.

[CR127] Yu W, Wang P, Ma H, Zhang G, Yulin Z, Lu B, Wang H, Dong M (2014). Suppression of T-type Ca2+ channels inhibited human laryngeal squamous cell carcinoma cell proliferation running title: roles of T-type Ca2+ channels in LSCC cell proliferation. Clin Lab.

[CR128] Yu XJ, Yang MJ, Zhou B, Wang GZ, Huang YC, Wu LC, Cheng X, Wen ZS, Huang JY, Zhang YD, Gao XH, Li GF, He SW, Gu ZH, Ma L, Pan CM, Wang P, Chen HB, Hong ZP, Wang XL, Mao WJ, Jin XL, Kang H, Chen ST, Zhu YQ, Gu WY, Liu Z, Dong H, Tian LW, Chen SJ, Cao Y, Wang SY, Zhou GB (2015). Characterization of Somatic Mutations in Air Pollution-Related Lung Cancer. Ebiomedicine.

[CR129] Zamponi GW, Striessnig J, Koschak A, Dolphin AC (2015). The physiology, pathology, and pharmacology of voltage-gated calcium channels and their future therapeutic potential. Pharmacol Rev.

[CR130] Zhu G, Liu Z, Epstein JI, Davis C, Christudass CS, Carter HB, Landis P, Zhang H, Chung JY, Hewitt SM, Miller MC, Veltri RW (2015). A novel quantitative multiplex tissue immunoblotting for biomarkers predicts a prostate cancer aggressive phenotype. Cancer Epidemiol Biomark Prev.

